# Academic Motivation of Students Experiencing Person-Environment Misfit in Social Work Educational Settings: The Role of Social Dominance Orientation

**DOI:** 10.3390/ejihpe14020018

**Published:** 2024-01-24

**Authors:** Alessio Tesi, Daniela Di Santo, Antonio Aiello

**Affiliations:** Department of Political Science, University of Pisa, Via F. Serafini, 3, 56126 Pisa, Italy; daniela.disanto@unipi.it (D.D.S.); antonio.aiello@unipi.it (A.A.)

**Keywords:** academic motivation, person-environment fit, self-determination, social dominance, social work

## Abstract

Interweaving social dominance, person-environment fit, and self-determination theories, the present study sought to understand whether the attrition between students’ levels of social dominance orientation and the hierarchy-attenuating function of the social work faculty in which they study may influence students’ academic motivational pathways. A total of 221 undergraduate social work students participated in the study and completed a self-report questionnaire. Participants’ social dominance orientation, person-environment misfit, and academic intrinsic and extrinsic motivation were measured. Results indicated that students’ social dominance orientation was associated with an external rather than an internal regulation of their academic motivation, mediated by their perceived person-environment misfit. For those students who personally support group-based inequalities, exposure to hierarchy-attenuating contexts would lead to regulating their academic behavior toward the pursuit of extrinsic (vs. intrinsic) goals, that is, studying to gain financial benefits and social prestige, in accordance with the pursuit of their beliefs of social dominance.

## 1. Introduction

To build a positive, fulfilling, and meaningful experience in organizations, people, wherever they work, study, or perform any activity in an organization, should perceive a sense of coherence, direction, and meaning between what they are (i.e., their self) and what is most supported, promoted, and shared (i.e., social norms and values) within the organization as a whole (i.e., organizational culture) [1]. Such compatibility between an individual’s personal characteristics and the social norms and values shared in an environment was defined as person-environment (P-E) fit [2,3]. The P-E fit has been operationalized according to the assessment of the compatibility between the person (P) factor, which includes the individual’s personality, skills, needs, goals, attitudes, values, ambitions, ideals, and preferences, and the environment (E) factor, which as a whole includes the organization’s shared social norms, social representations of careers, and how relationships and roles are explicitly and implicitly handled in a given environment [3,4]. Although a significant amount of research has focused on investigating the role of P-E fit as one of the most important predictors of well-being in work environments (i.e., motivations, performance, and job satisfaction) [4], few studies have explored the possible consequences of the opposite condition, called P-E misfit [5], that is, the perceived attrition between the “P” and “E” factors. 

In the present study, we entered this line of research on P-E misfit with the aim of investigating how people’s motivations would be shaped by a particular form of socio-political P-E misfit resulting from an attrition between a personal orientation towards the opposition of equality and an organizational culture supporting inclusion and egalitarianism [6]. It is worth noting that different types of P-E misfits can represent different types of attrition [7]. Specifically, a misfit can result from the incompatibility between a person’s characteristics and the expected tasks of his or her job (i.e., person-job misfit), from the mismatch between the person’s attitudes and those espoused by his or her restricted group of colleagues (person-group misfit), or even from values incompatibility [8]. 

In particular, we build on a recent line of research [6,9,10] that found that people’s social dominance orientation (SDO; the extent to which one supports group-based hierarchies and inequalities) is related to perceived P-E misfit in social work settings [11], which are known to be hierarchy-attenuating environments characterized by a strong prevalence and support of social norms that oppose dominance and value social norms of inclusion and equality [11,12]. As a possible consequence of such P-E misfit, scholars [10] found that people who perceive P-E misfit can adopt two possible strategies to deal with such attrition: (i) they can choose to drop out of the context or (ii) they can devalue the helping and hierarchy-reducing pro-social mission of social work in favor of pursuing self-enhancement goals (i.e., studying social work to gain more money or success). This focus on self-enhancement motives would guarantee people a reduction in the dissonance associated with engaging in an environment that does not fit with their dominant mindset. However, knowledge about the motivational patterns of those who perceive a P-E misfit is still limited, and thus more evidence is needed. 

The present study aims to investigate whether the incompatibility between individuals’ SDO and a hierarchy-attenuating organizational culture may influence motivational pathways. In particular, we expected that people who perceived a P-E misfit would be driven by an extrinsic motivation focused on goals of self-enhancement, which may detract from the helping mission pertaining the social work practice. Therefore, we recruited a sample of undergraduate social work students (i.e., a well-recognized hierarchy-attenuating environment) [9,11,13] to examine the possible effects of SDO and the P-E misfit on their academic motivation. Following previous research [14], we draw on a widely used motivational theory in education, namely self-determination theory [15,16,17,18,19]. Specifically, we linked social dominance theory, P-E fit theory, and self-determination theory, hypothesizing that the P-E misfit perceived by students who endorse SDO and inhabit a hierarchy-attenuating context may be associated with the trend of their academic trajectory by engrossing their focus on extrinsic (vs. intrinsic) motivation. These extrinsic motives include the desire to receive a higher salary or to be recognized as a successful person [16], with the consequence of potentially exploiting a hierarchy-attenuating context for self-enhancing hierarchy-enhancing motives.

## 2. Theoretical Background

### 2.1. Social Dominance Theory and P-E Misfit

Social dominance theory is a widely recognized theoretical framework that aims to explain the production and reproduction of social inequalities through processes involved in the maintenance of intergroup asymmetries [20,21]. According to social dominance theory, people endorse legitimizing myths of hierarchies (i.e., stereotypes, prejudices, racism, sexism, belief systems supporting meritocracy, and the death penalty) differently based on their levels of SDO, that is, a personal orientation capturing the extent to which people tend to support group-based hierarchies and inequalities [20,21]. 

Social norms and ideologies shared in specific contexts can also enhance or attenuate social hierarchies [13]. Hierarchy-enhancing contexts (e.g., military and profit-maximizing organizations) advocate and promote a culture of group-based inequalities through a differential distribution of power (social, political, and economic) in favor of dominant groups; in contrast, hierarchy-attenuating contexts (e.g., humanitarian, nonprofit, and social work organizations) advocate and promote egalitarian social norms that prioritize reducing group-based inequalities [13]. A number of studies [12,22,23,24] highlight that people higher in SDO report a greater P-E fit in hierarchy-enhancing contexts and are more likely to pursue hierarchy-enhancing careers (i.e., police); in contrast, low SDO people report greater fit in hierarchy-attenuating environments and are more likely to pursue hierarchy-attenuating careers (i.e., social work). Consistently, people higher in SDO also report greater P-E misfit in hierarchy-attenuating contexts [9,11]. 

Even in academic contexts, students generally evaluate the congruence between their SDO and the values embodied in their academic environment’s culture. According to Haley and Sidanius [13], people are generally more willing to be engaged in environments that promote the same social norms and values they personally endorse. Thus, it is plausible to suppose that people enrolling in social work studies have a similar value system as promoted by the social work educational context. However, as also found in previous studies [9,10], SDO levels may vary in people enrolled in these courses, and consequently, the fit/misfit is experienced. Therefore, it is interesting to study the motivations and career outcomes of those who experience an attrition with the context, that is, a P-E misfit [8,9]. Studies show that P-E misfit has generally negative consequences for students’ quality of life, such as increasing the likelihood that they will drop out of the academy [10]. Scholars [10] also found evidence that social work students’ levels of SDO were associated with P-E misfit toward the egalitarian social norms promoted in the context; P-E misfit, in turn, fostered students’ intentions to neglect the social work mission (i.e., helping others in disadvantaged positions) in pursuit of goals favoring competitiveness and self-enhancement [14,25,26]. This preliminary evidence suggests that the P-E misfit experienced by those who endorse SDO and inhabit hierarchy-attenuating environments may influence people’s focus on extrinsic rather than intrinsic forms of motivation.

### 2.2. Self-Determined Motivation and SDO

Self-determination theory [15,16] is one of the most influential theoretical frameworks for studying human motivation. Self-determination theory highlights the role of social, environmental, and cultural factors in facilitating or undermining people’s sense of will and initiative. According to self-determination theory, the socio-environmental conditions supporting psychological needs of autonomy, competence, and relatedness would fulfill broad people’s desire for personal growth, resulting in an increased commitment to activities and consequent performance improvement [15,16,27]. On the other hand, conditions that hinder the satisfaction of these basic psychological needs would impair performance and well-being [27,28,29]. According to self-determination theorists, any behavior can be motivated intrinsically or extrinsically [15,16,30]. Intrinsic motivation is defined as engaging in an activity for its inherent pleasure and satisfaction rather than for an external reward [15,31]. In contrast, extrinsic motivation refers to doing something not for its inherent pleasure but for a separable outcome (e.g., an external reward or avoidance of punishment). The continuum between extrinsic and intrinsic motivation can be further distinguished into external, introjected, identified, and internal regulations ranging from the lowest to the highest level of internalization of motivation [30]. In particular, external regulation represents the lowest level of internalization, referring to behavior controlled by external sources (e.g., demands and pressures). Introjected regulation indicates a behavior that is controlled by the person’s internal demands (e.g., a sense of obligation or self-esteem contingencies such as self-enhancement). Identified regulation refers to behaviors chosen because they are valued as important by the individual, even in relation to the context in which he or she operates [30]. Finally, internal regulation is the act of engaging in a behavior because it is genuinely pleasurable to the individual. Intrinsic and identified regulations may be classified as ‘autonomous’ motivation (i.e., focused on an internal regulation and a full sense of volition). In contrast, identified and extrinsic regulations can be classified as ‘controlled’ motivations since they focus more on the pressure to pursue external goals (i.e., higher salary and fame) [32].

Another way to synthesize the external–internal continuum of motivational regulation is to use the relative autonomy index (RAI) [19,30,33]. Once the scores of the different self-determined regulations are measured with the specific self-report scales, it is possible to calculate a combined score (i.e., the RAI) that weights and combines the scores of each motivational regulation scale with a specific formula (RAI = (−2 × External) + (−1 × Introjected) + (1 × Identified) + (2 × Internal)). Specifically, the RAI measures how much a person’s motivation is autonomously regulated internally, that is, it determines a person’s position on the continuum of external–internal regulation with regard to achieving a specific goal. A higher score on RAI indicates internal regulation, while a lower score indicates a greater focus on external regulation.

The validity of self-determination theory in education is widely supported [17,18]. Different self-determined motivations can significantly impact individual performance, choices, and educational outcomes [16,29]. For example, the choice to persist or drop out of the school context is regulated by a more autonomous than controlled regulation of academic motivation [33]. Regarding possible determinants of self-determined motivation, studies found that academic motivation could be influenced by students’ self-perceptions of autonomy and scholastic competence, driven by socially supportive behaviors [18]. 

As far as the role of SDO in influencing self-determined motivation, Duriez and colleagues [14] found evidence for a reciprocal relationship between extrinsic–intrinsic motivation and SDO. Specifically, they found that extrinsic (vs. intrinsic) goal pursuit predicted SDO, but SDO also predicted extrinsic (vs. intrinsic) goal pursuit. These findings suggest that extrinsic motivation and SDO are reciprocally related. Since people higher in SDO aim to achieve, maintain, or foster their social dominant position in a given context, they would be more likely to focus on extrinsic (vs. intrinsic) goals (i.e., attain economic resources and social prestige), with the aim of fostering their hierarchal placement [14]. According to this notion, it has been found that the more people report high levels of SDO, the more they are willing to support harsh influence tactics as external means to enhance or maintain hierarchies in organizations, and this occurs especially in hierarchy-attenuating contexts where people with higher levels of SDO do not fit into the organizational culture [34]. We expected that perceived attrition in a hierarchy-attenuating environment would increase people’s willingness to focus on external regulation of motivation. Accordingly, we sought to further explore the relationship between SDO and self-determined motivation in a hierarchy-attenuating educational context by examining whether SDO levels would influence social work students’ academic motivation via perceived P-E misfit. 

## 3. The Present Study

Social work educational settings are characterized by promoting social norms to reduce social inequalities [9,11]. Therefore, we would expect social work students’ SDO to be associated with introjected and external regulation of their academic motivation because these forms of self-determined regulation are more related to the goals of pursuing financial success and self-enhancement—goals that are more akin to their endorsement of group-based inequalities [10]. Consistently, we would expect them to harbor lower levels of internal regulation and identified regulations because these forms of regulation are more related to the intrinsic enjoyment of studying for a career in social work. Studying social work to achieve external goals mostly related to self-enhancement may increase the risk of devaluing the social work mission, which instead focuses on strengthening the health and social conditions of people living in vulnerable circumstances (e.g., immigrants, people with disabilities, and elders) [35]. Considering this, we hypothesized that:

**H1.**  Social work students’ SDO was (a) positively correlated with introjected and external regulation and (b) negatively correlated with internal and identified regulation.

**Figure 1:**
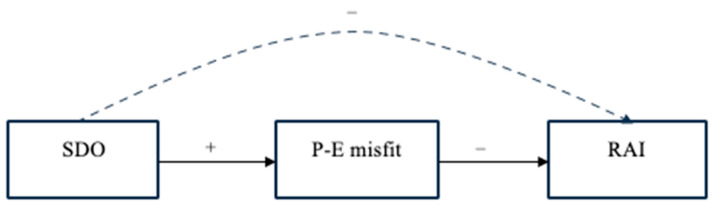
The study’s model (hypothesis 2). Note. SDO = social dominance orientation; P-E misfit = person-environment misfit; RAI = relative autonomy index. Continuous lines represent direct effects, and the discontinuous line indicates the indirect effect.

This research also aims to advance the previous literature [10] by proposing that the P-E misfit of social work students, resulting from their personal endorsement of SDO, would trigger their decision to rely on extrinsic (vs. intrinsic) goal pursuit. Specifically, we hypothesized that (i) social work students’ SDO would be associated with a greater P-E misfit toward the hierarchy-attenuating context, which in turn (ii) might lead students to have a negative RAI, i.e., to focus the pursuit of their academic careers more on extrinsic motivation, which is aligned with the SDO’s desire for self-improvement within social hierarchies, rather than intrinsic motivation, which is more aligned with an interest in the helping mission promulgated in the social work context (i.e., mitigating social hierarchy by improving the position of vulnerable people). In sum, we hypothesized (Figure 1):

**H2.**  The negative association between social work students’ SDO and RAI was mediated by the P-E misfit.

## 4. Methods

### 4.1. Participants and Procedure

We conducted a cross-sectional study recruiting a convenience sample of Italian students enrolled in a social work program. Social work faculties are considered hierarchy-attenuating environments to the extent that they promote and share hierarchy-attenuating legitimizing myths about the scope of social work to reduce poverty and inequality and improve the conditions of disadvantaged people [6,9,11].

To estimate the adequate sample size for testing the mediation mode, we used the Monte Carlo power analysis tool for indirect effects [36]. According to the coefficients reported in previous literature [6,9], we set moderate effect sizes (all paths, r = 0.30), 5.000 replications, and 20.000 Monte Carlo draws per replication. The analysis suggested a minimum of 160 participants to reach a sufficient power of 0.80 for the indirect effect. 

We initially contacted a total of 250 social work students. Ultimately, 221 students voluntarily agreed to participate in this research (response rate of 88.40%). Participants were 207 females and 14 males (163 bachelor’s degree students and 58 master’s degree students), and their mean age was 22.54 years (SD = 3.75). The research was conducted according to the guidelines of the Declaration of Helsinki. Participants provided explicit, informed consent to participate and anonymously filled out an online survey. 

### 4.2. Measures

#### 4.2.1. Social Dominance Orientation

SDO was assessed through the Italian adaptation [37] of the Social Dominance Orientation Scale_7_ [21]. The scale measures people’s support for group-based hierarchies and inequalities and comprises 16 items (i.e., “An ideal society requires some groups to be on the top and others on the bottom”), with a 7-point Likert response format ranging from 0 = “strongly disagree” to 6 = “strongly agree”. In the present study, the scale presented adequate internal consistency (Cronbach’s alpha = 0.86).

#### 4.2.2. Person-Environment Misfit 

P-E misfit was assessed through the Italian adaptation [10] of the scale proposed by Deng and colleagues [38]. The scale measures the P-E misfit by asking individuals to indicate the extent to which their personal values are incompatible with the values promoted in the social work context. The scale consists of three items (i.e., “The things I value in life are not similar to the things the social work major values”, “My personal values do not match the culture of the social work major”, “The values and culture of the social work major do not provide a good fit with the things I value in life”) with a 7-point Likert scale response format ranging from “0 = strongly disagree” to 6 = “strongly agree”. In the present study, the scale showed sufficient internal consistency (Cronbach’s alpha = 0.69). In accordance with the literature [5,8], the accessibility of the values and social norms related to social work was facilitated for the participants through a priming procedure [9]. Prior to completing the P-E misfit scale, participants read the following statement:

“A number of studies have indicated that social work programs are among those that have as a core value the reduction of social hierarchies and inequalities among groups. In particular, the policies and practices taught in social work programs promote the empowerment of socially, politically, and economically disadvantaged people (e.g., immigrants, the poor, the elderly, and the disabled) in an attempt to reduce the disparities between these people and those with privileges. In light of what you have read above, please respond to the statements in the questionnaire by indicating how much you agree or disagree with each statement.”

#### 4.2.3. Academic Motivation 

To assess students’ motivation according to self-determination theory, the Italian adaptation [33] of the Academic Motivation Scale [39] was used. Specifically, four types of regulation related to students’ academic motivation were assessed on a continuum ranging from external, introjected, identified, and intrinsic regulation. We excluded the measure of amotivation because it implies a complete absence of self-determined motivation [18]. Because university students can autonomously choose a particular academic path, some form of external or internal self-determined motivation is involved in sustaining their choices and academic behaviors. The scale comprises 16 items that capture the reasons for pursuing a degree in social work: external regulation (i.e., “In order to obtain a more prestigious job later on”), introjected regulation (“Because I want to show myself that I can succeed in my studies”), identified regulation (“Because this will help me make a better choice regarding my career orientation”), and intrinsic regulation (“For the pleasure that I experience in broadening my knowledge about subjects that appeal to me”). Participants responded on a 5-point Likert scale ranging from 1 = “Does not correspond at all” to 5 = “Corresponds exactly”. In the present study, the scales presented adequate internal consistency, that is, external regulation, Cronbach’s α = 0.84; introjected regulation, Cronbach’s α = 0.81; identified regulation, Cronbach’s α = 0.92; and intrinsic regulation, Cronbach’s α = 0.85.

## 5. Results

### 5.1. Descriptive Statistics and Correlations

Descriptive statistics and bivariate correlations are reported in Table 1. As shown, SDO is significantly and positively correlated with perceived P-E misfit in the considered setting. 

Consistent with our hypothesis, SDO is significantly and positively correlated with external and introjected regulations, significantly and negatively correlated with identified regulations, and marginally significantly and negatively correlated with internal regulations (*p* = 0.06). In accordance with our hypothesis, SDO was also significantly and negatively correlated with the participants’ RAI.

A similar pattern was found for the perceived P-E misfit; the P-E misfit significantly and positively correlated with external regulation and significantly and negatively correlated with identified and intrinsic regulations and with the participants’ RAI. 

Concerning the correlations between the self-determined regulations, external and introjected regulations significantly and positively correlated with each other, identified and intrinsic regulations significantly and positively correlated with each other, and identified regulations significantly and negatively correlated with both external and introjected regulations. 

### 5.2. Mediation Model

**Figure 2:**
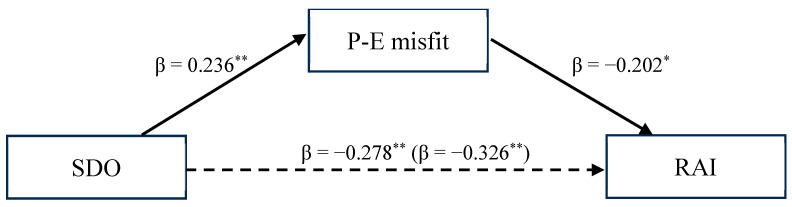
A mediation model showing the effects of SDO on the relative autonomy index via the P-E misfit. Note: single-starred associations are significant at the *p* ≤ 0.01 level, and double-starred associations are significant at the *p* ≤ 0.001 level. SDO = social dominance orientation; P-E misfit = person-environment misfit; RAI = relative autonomy index. The total effect is inside the parentheses. The discontinuous line indicates that a significant indirect effect of SDO on the RAI was found.

We tested our hypothesized mediation model through mediation analysis (PROCESS Macro; Model 4) [40] with a 95% CI and 5000 bootstrap samples. Results are presented in Table 2 and Figure 2. In Table 2, both standardized and unstandardized regression coefficients are reported. 

SDO was positively and significantly associated with perceived P-E misfit in the considered context (*β* = 0.236 and *p* < 0.001). In turn, the perceived P-E misfit was negatively and significantly associated with RAI (*β* = −0.202 and *p* = 0.002). 

The total effect of SDO on RAI was negative and significant (*β* = −0.326, *b* = −1.459, *SE* = 0.286, 95%CI [−2.023, −0.895], and *p* < 0.001). Importantly, the indirect effect of SDO on RAI via the perceived P-E misfit was also significant, confirming our mediation hypothesis (completely standardized indirect effect = −0.048, BootSE = 0.023, and 95% BootCI [−0.102, −0.011]).

## 6. Discussion

The present study aimed to deepen how the P-E misfit [5,7,8] between social work students’ SDO and the normative setting consisting of hierarchy-attenuating social norms can potentially elicit students’ extrinsic vs. intrinsic motivation.

In line with the recent literature [6,9], we found that social work students’ SDO was associated with the P-E misfit with the educational context. Previous studies [9,10] have also found that social work students who had higher levels of SDO reported a greater willingness to leave the educational environment, highlighting that one of the viable solutions to address P-E attrition toward the context (i.e., P-E misfit) is to choose a “leaving” strategy; however, dropping out entails a series of costs for both students and the educational environment in terms of loss of economic, psychological, and social resources. Consequently, some students may choose to “persist” in a misfitting environment but adopt a set of motivations and goals consistent with their SDO mindset. According to this notion, our results highlighted that students’ SDO was negatively related to intrinsic and identified regulation of their academic motivation and positively related to introjected and external regulation. Therefore, in line with previous results [14], we found that the more students endorse group-based asymmetries (i.e., SDO), the more their academic behavior is regulated by external goals that potentially advance their position in social hierarchies (i.e., higher salary and social prestige), rather than being motivated by the enjoyment of pursuing a social work career [22]. Notably, consistent with our findings, previous studies found that SDO motivates people to endorse harsh social influence tactics as a means of achieving or maintaining stable social hierarchies. Furthermore, and consistent with less pursuit of intrinsic goals related to social work, higher levels of SDO have been found to be positively associated with a competitive worldview [25], a desire for self-enhancement, achievement, and power [41]. Previous research has also found a negative association between SDO and empathy for low-status groups [26], self-transcendence, universalism, and benevolent values [42].

The present study advances the literature [10,14] by showing that SDO is associated with an external (vs. internal) regulation of motivation via a perceived incompatibility (i.e., P-E misfit) with the environment. The occurrence of P-E misfit— a perceived psychological distance from hierarchy-attenuating values — leads students to pursue academic goals for their own interest, consistent with their SDO. Further, P-E misfit may impede an authentic connection to the hierarchy-attenuating cultural assortment promoted by a social work educational setting [10,13,24] and thus the likelihood of engaging in such a career for its inherent enjoyment (i.e., intrinsic motivation). 

### 6.1. Practical Implications for Social Work Educational Settings

The pursuit of extrinsic academic motivation may have consequences for social work students [10]. External regulation of motivation has been associated with cognitive (i.e., low concentration, attention, and memory), affective (i.e., less interest, satisfaction, and positive emotions), social (i.e., low quality of interpersonal relationships), and behavioral (i.e., intensity and performance) consequences [18,19]. In addition, low internal regulation of motivation exposes students to a range of negative lifelong outcomes, including low educational attainment, low achievement and engagement, low academic retention, and a higher likelihood of dropping out of the educational setting [29,31,43].

Taken together, our findings underscore that students’ SDO would threaten their internalization of social work principles of support and assistance to vulnerable groups [35]. This would affect students’ outcomes and their persistence in academic tasks [19]. In addition, we found support that the academic behavior of social work students experiencing SDO-related P-E misfit would be sustained more by pro-self than pro-social motivations [10]. This evidence may provide insight into the career path of social work students [22]. For example, engaging in the social work profession by pursuing self-enhancement motivations could imply a derogation from the social work mission, exposing social workers to the risk of stress or burnout and jeopardizing the quality of services provided [10,11]. Therefore, social work educational institutions should pay attention to the implementation of orientation programs for incoming students by preventing students’ attrition towards social work norms and principles and establishing the conditions for an optimal P-E fit with positive consequences for their work passion [44]. Creating an educational environment that welcomes diverse perspectives, visions, and attitudes could be beneficial to foster an aware university community of practice that facilitates the exchange of ideas, creativity, and the development of collaborative solutions and shared meanings in social work practice [45]. 

### 6.2. Limitations and Future Perspectives

The limitations of the current research could be addressed in future studies. In particular, its cross-sectional nature hinders the interpretation of the relationships between variables in terms of causality. Related to the cross-sectional design of the present study, future studies should also employ a longitudinal design to examine whether a misfit experienced over time can further alter an individual’s motivational pattern, for example, by decreasing intrinsic motivation or increasing extrinsic motivation. Another interesting longitudinal line of research could be devoted to examining the role of social influence in shaping students’ decisions [46] to enroll in a social work major and in the persistence of students’ academic motivation over time. For example, future studies could test whether students who are extrinsically motivated prior to enrolling in a particular major (i.e., conforming to the expectations of others) can replicate this pattern over time (i.e., during the course of study) and whether the degree of SDO and P-E fit or misfit can enhance their external regulation of motivation. Although further studies are necessary to grasp the intricacies of causal relationships among the variables under study, it is worth mentioning that our mediational model relies on prior evidence supporting the role of SDO as an antecedent of P-E misfit and intrinsic/extrinsic motivation [9,14]. 

Moreover, caution must be exercised when extending the results of the present study to other hierarchy-attenuating settings. For instance, future studies could evaluate our model among practicing social workers. An intriguing line of research may involve the deepening of which variables can drive the intention to drop out or persist as a consequence of a P-E misfit. For example, our model could be enriched by integrating measures of intention to leave or place attachment. In this way, it might be possible to compare the motivational patterns of people who are more inclined to “leave” with people who decide to “stay” in the study/work setting despite the perceived attrition. In addition, future research could examine the potential consequences of endorsing external motivation (vs. internal motivation) based on SDO levels. It would be interesting to assess whether the discrepancy between the person (i.e., SDO levels) and the environment (i.e., hierarchy-attenuating social norms) hinders the perceived well-being of students, professionals, and service users.

## 7. Conclusions

The present study extends previous findings [10,14] by highlighting that the attrition (i.e., P-E misfit) between social work students’ levels of SDO and the social norms promoted and shared in social work settings leads to external rather than internal motivations to pursue an academic career. These findings are particularly relevant to understanding the role of SDO in shaping the motivation of people inhabiting hierarchy-attenuating educational contexts [13], highlighting that those who experience a P-E misfit are more likely to pursue self-enhancement goals according to their dominance beliefs [47]. Further research could investigate whether individuals who endorse SDO and persist in a social work environment can utilize the hierarchy-attenuating setting for self-enhancement objectives and what the implications may be for the long-term feasibility of social work practice. 

## Figures and Tables

**Table 1:** Bivariate correlations and descriptive statistics.

	1	2	3	4	5	6	7	M (SD)
1. SDO	(0.86)							1.070 (0.782)
2. P-E misfit	0.236 **	(0.69)						1.208 (1.022)
3. External regulation	0.251 **	0.144 *	(0.84)					2.519 (1.084)
4. Introjected regulation	0.223 **	0.022	0.503 **	(0.81)				2.187 (1.012)
5. Identified regulation	−0.260 **	−0.365 **	−0.151 *	−0.166 *	(0.92)			4.622 (0.638)
6. Intrinsic regulation	−0.128 ^+^	−0.229 **	0.031	0.038	0.501 **	(0.85)		4.141 (0.807)
7. Relative autonomy index	−0.326 **	−0.268 **	−0.778 **	−0.613 **	0.554 **	0.522 **	-	5.680 (3.501)

Note: ^+^ *p* = 0.06, * *p* ≤ 0.05, and ** *p* ≤ 0.001. In the bracket is Cronbach’s alpha. *N* = 221.

**Table 2:** P-E misfit and RAI regressed on SDO.

P-E Misfit							
		β	B	SE	95% CI		p
					LL	UL	
SDO		0.236	0.309	0.086	0.140	0.478	<0.001
	R = 0.236 and R^2^ = 0.056						
RAI							
		β	B	SE	95% CI		p
					LL	UL	
SDO		−0.278	−1.245	0.289	−1.814	−0.677	<0.001
P-E misfit		−0.202	−0.691	0.221	−1.126	−0.256	0.002
	R = 0.380 and R^2^ = 0.145						

## Data Availability

The data presented in this study are available on request from the corresponding author.
